# Prevalence, Virulence Potential, and Growth in Cheese of *Bacillus cereus* Strains Isolated from Fresh and Short-Ripened Cheeses Sold on the Italian Market

**DOI:** 10.3390/microorganisms11020521

**Published:** 2023-02-18

**Authors:** Erica Tirloni, Cristian Bernardi, Francesco Celandroni, Diletta Mazzantini, Mariacristina Massimino, Simone Stella, Emilia Ghelardi

**Affiliations:** 1 Department of Veterinary Medicine and Animal Sciences, University of Milan, Via dell’Università 6, 26900 Lodi, Italy; 2 Department of Translational Research and New Technologies in Medicine and Surgery, University of Pisa, Via San Zeno 37, 56127 Pisa, Italy; 3 Research Center Nutraceuticals and Food for Health-Nutrafood, University of Pisa, 56128 Pisa, Italy

**Keywords:** *Bacillus cereus*, cheese substrate, prevalence, pathogenic potential, growth in cheese, toxins, biofilm

## Abstract

This study investigated *B. cereus* presence in 122 samples belonging to 34 typologies of fresh or short-ripened cheeses made from cow, sheep, goat, or buffalo pasteurized milk, and sold on the Italian market. *B. cereus* was isolated at a prevalence of 9.8%, with a marked variability among cheese categories, and at low counts (always below 2.26 Log CFU/g). Twelve isolates were identified by MALDI-TOF analysis and typified by RAPD PCR as belonging to different *B. cereus* strains. All the strains were tested for the production of hemolysin BL, phosphatidylcholine-specific phospholipase C, proteases, and biofilm formation, and for the presence of chromosomal toxin-encoding genes (*sph*, *plcA*, *cytK*, *entFM*, *bcet*, *nheA*, *nheB*, *nheC*). Overall, 92% of strains harbored *bcet*, 75% the three genes *nheA*, *nheB*, and *nheC,* as well as *plcA* and *sph*, 67% *entFM*, and 33% *cytK*. All strains showed biofilm-forming ability. A chemical-physical characterization of the cheeses was also performed to show their suitability as substrates for *B. cereus* growth, showing high heterogeneity in terms of pH, aw, salt content, and concentration of organic acids. Finally, the ability to support spore germination and vegetative cell growth of a selected cheese was investigated in spores-inoculated samples maintained at 10 °C and 15 °C, showing the inhibitory effect of low storage temperatures.

## 1. Introduction

*Bacillus cereus* is a rod-shaped, aerobic or facultative anaerobic, motile, and spore-forming microbe ubiquitously distributed in the environment, including soil, water, and decaying materials, and is frequently isolated as a food contaminant. In humans, *B. cereus* is primarily responsible for food-borne illnesses, which are caused by the ingestion of foods contaminated with living bacteria/spores, toxins, or both. In addition, the microbe can behave as an opportunistic pathogen able to determine local and systemic infections [1,2]. *B. cereus* pathogenicity is due to the production of several virulence proteins and enzymes, including hemolysin BL [HBL], non-hemolytic enterotoxin [NHE], cytotoxin K [CytK], enterotoxin T [Bcet], enterotoxin FM [EntFM], phosphatidylcholine-specific phospholipase C [PC-PLC], sphingomyelinase (Smase), phosphatidylinositol-specific phospholipase C [PI-PLC], and proteases [3].

On dairy farms, *B. cereus* can be isolated at all stages of the production chain. At farm level, its presence and diffusion are mainly due to soil, fodder, and bedding contamination. In fact, these materials are in direct contact with cows and can behave as carriers of the bacterium. At the time of milking, spores of *B. cereus* can be easily transferred to milk. Their subsequent inactivation by thermal treatments depends on the time/temperature combination. For the production of most cheeses and other dairy products, milk is used raw, or submitted to thermization or pasteurization; such processes cannot ensure a complete inactivation of the spores. Conversely, these thermal processes can act as activators of spore germination [4]. Once germinated, vegetative cells of *B. cereus* can replicate in dairy products when favorable environmental conditions, depending on growth substrate (pH, aw, salt content) and extrinsic parameters (e.g., temperature), are established. In addition, *B. cereus* can directly produce virulence proteins and enzymes in dairy products, thus causing the spoilage of the product and constituting a potential health risk for consumers.

The dairy production system is an extremely complex environment with multiple sources of contamination. In addition, cleaning procedures of dairy plants may be extremely challenging due to the presence of dead ends, pockets, and traps. In these structures, bacteria like *B. cereus* can find an advantageous niche where spores can adhere, persist resisting sanitization procedures, and germinate, causing post-processing contamination of milk and milk products [5]. Moreover, the ability of *B. cereus* to produce biofilm creates an additional chance for the microorganism to survive in hostile environments and may favor contamination during the dairy production process.

Many studies have evaluated *B. cereus* prevalence in milk and milk products, showing heterogeneous results. In fact, in raw milk, the prevalence of the microbe ranged from 3.8 to 100%, with a microbial load up to 5 Log cfu/mL. Prevalence from 2 to 65.3% was reported in pasteurized milk, with a microbial concentration up to 5 Log cfu/mL. Various studies have reported prevalence from 6.8 to 68% in milk powder and infant formulas, with bacterial load up to 4 Log cfu/g. Focusing on cheese, *B. cereus* showed prevalence from 0 to 95% in products depending on the production process applied and the ripening duration, with concentrations up to 6 Log cfu/g [6,7,8,9,10]. 

Some studies have investigated the growth potential of *B. cereus* in milk and milk products, with different outcomes determined depending on the presence of hurdles that limit/inhibit the replication of the microorganism (e.g., chemical-physical characteristics of the substrates, natural microflora) [11,12].

The aims of this study were to: (i) investigate the prevalence of *B. cereus* in fresh and short-ripened cheeses sold on the Italian market; (ii) establish if some particular strains are more recurrent in cheeses; (iii) determine if contaminating strains may be potentially pathogenetic; (iv) evaluate if some selected strains are experimentally able to grow in a cheese matrix at different temperatures.

## 2. Materials and Methods

### 2.1. B. cereus Prevalence in Cheese

A total of 122 fresh or shot-ripened cheese samples were obtained from the Italian market. The samples were obtained through 7 sampling sessions, performed at large retail stores belonging to different distribution brands to obtain a broad picture of a section of the market. During each session, all the different products that met the study requirements (fresh cheeses or short-ripened cheeses, soft cheeses ripened for a maximum period of 50 days) were taken, avoiding repeated samplings on the same product. The samples belonged to 34 different typologies, 21 of which derived from cow milk, 7 from goat milk, 2 from sheep milk, 2 from buffalo milk, 1 from mixed sheep/goat milk, and 1 from mixed cow/goat milk. All the cheeses were obtained from pasteurized milk. The sampling protocol included 4 typologies of “pasta filata” (spun paste) cheeses, 11 of other fresh cheeses, 3 of ricotta cheese (made from whey), 1 of mascarpone cheese (made from milk cream), and 15 of short-ripened cheeses, 6 of which with the addition of molds. The samples were immediately transported to the laboratory and analyzed the day of purchase. The presence of *B. cereus* was investigated according to ISO 7932:2004 [13]: briefly, 10 g of product was diluted 10-fold in chilled sterile diluent solution (0.85% NaCl and 0.1% peptone) and homogenized for 60 s in a Stomacher 400 (Seward Medical, London, UK). Then, appropriate 10-fold dilutions of the homogenates were made in chilled saline. *B. cereus* was enumerated by spreading aliquots onto PEMBA agar (Scharlab, Barcelona, E) and incubating at 30 °C for 48 h. For the enumeration of spores, homogenates were thermally treated at 80 °C for 10 min in order to kill vegetative cells before plating and seeded as above. Presumptive *B. cereus* colonies were picked from the plates (up to five colonies for each positive sample) and maintained at −80 °C in Microbank Cryogenic vials (Pro-Lab Diagnostics U.K., Merseyside, UK).

### 2.2. Identification of B. cereus by MALDI-TOF MS

MALDI-TOF MS identification was performed as previously reported [14,15]. Briefly, well-isolated colonies (up to five for each positive sample) were spotted on the MALDI plates and covered with 1 µL of ethanol, 1 µL of formic acid, 1 µL of acetonitrile for lysis, and were overlayed with 1 µL of saturated α-cyano-4-hydroxycinnamic acid (HCCA) matrix solution, accordingly to manufacturer specifications. Plates were air-dried and placed in the instrument according to the manufacturer’s instructions. The mass spectra were automatically acquired within 10 min, in the positive linear mode at a laser frequency of 60 Hz with an acquisition range from 1960 to 20,000 Da. Spectra were imported into the integrated MALDI Biotyper software (version 3.1, Bruker, Billerica, MA, USA) and analyzed by standard pattern matching with a default setting. A score of ≥2.00 indicated identification at the species level; a score ranging from 1.99 to 1.70 indicated identification at the genus level; whereas any score < 1.70 meant no significant similarity of the obtained spectrum with any database entry [15]. Each isolate was tested in triplicate. 

### 2.3. RAPD-PCR

Genomic DNA was extracted from *B. cereus* isolates as previously described [16]. RAPD-PCR fingerprinting of bacterial genomes was performed with the primers RPO2 (50-GCGATCCCCA-30), M13 (5-GAGGGTGGCGGCTCT-3), HLWL85 (50-ACAACTGCTC-30), and OPE03 (50-CCAGATGCAC-30). RAPD-PCR reactions were carried out in 50-µL mixtures containing 1 µM primer, 10 µL of Wonder Taq Reaction Buffer, 2.5 U of Wonder Taq (Euroclone, Milan, Italy), 50 ng of genomic DNA, and sterile ultrapure water up to 50 µL. PCR conditions were set as follows: 30 cycles consisting of 94 °C for 1 min, 36 °C for 1 min, and 72 °C for 2 min, followed by one cycle consisting of 72 °C for 10 min. Genomic DNA extracted from *B. cereus* ATCC 14579 and *B. cereus* ATCC 10987 were used as positive controls for each reaction. The reproducibility of RAPD-PCR profiles was assessed in at least three separate experiments. Clustering based on UPGMA was performed and dendrograms were applied with the Sørensen-Dice coefficient.

### 2.4. Detection of HBL, PC-PLC, and Proteases

The ability to produce and secrete hemolysin BL was assessed by streaking bacterial cells on sheep blood agar (Columbia agar containing 5% sheep-blood, Oxoid). Plates were incubated at 30 °C for 18 h. HBL secretion was tested by observing the formation of the typical discontinuous zone of hemolysis surrounding colonies [17]. The production of PC-PLC was evaluated by agar-diffusion assays by using 0.15% l-α-phosphatidylcholine (Sigma-Aldrich, Milan, Italy). Protease secretion was checked by seeding bacterial cells on 1.5% skim milk (Oxoid, Basingstoke, UK), followed by incubation at 37 °C for 18 h [18]. The presence of a clear degradation halo around colonies was indicative of the presence of proteolytic activities. Experiments were repeated three times on separate days. 

### 2.5. Detection of Toxin-Encoding Genes

For the detection of *B. cereus* toxin-encoding genes (Smase, *sph*; enterotoxin Bcet, *bcet*; enterotoxin FM, *entFM*; phosphatidylinositol-specific phospholipase; PI-PLC, *plcA*; cytotoxin K, *cytK*; NHEA, *nheA*; NHEB, *nheB*; and NHEC, *nheC*), PCR reactions were performed on bacterial genomic DNA. For each gene, primer pairs and amplification conditions were set as previously described [15]. 

### 2.6. Biofilm Formation

Isolates were tested for biofilm formation in Luria Bertani (LB) broth (Oxoid, Basingstoke, UK) as previously described [19]. Briefly, bacteria were grown to the early stationary phase in LB at 37 °C and diluted to an optical density at 600 nm (OD_600_) of 0.01 in fresh LB; 2 mL was transferred to wells of polystyrene 24-well plates (Falcon/Becton Dickinson, Franklin Lakes, NJ, USA) and incubated in static conditions for 48 h at 37 °C. At the end of incubation, non-adherent planktonic bacteria were removed and wells were washed three times with phosphate buffer saline (PBS) and air-dried. Microbial biofilms were stained with 2 mL of 0.3% crystal violet for 10 min, washed with distilled water, and air-dried. Crystal violet was solubilized with 2 mL of 70% ethanol and the OD590 was measured. The strong biofilm producer *B. cereus* ATCC 10987 was used as the positive control, while sterile LB was used as the negative control. Experiments were repeated three times on separate days and two technical replicates were carried out for each assay.

### 2.7. Chemical Physical Characterization of Cheese Samples

For each cheese sample, moisture [20], water activity (aw; Rotronic Hygromer Aw-DIO, Basserdorf, CH), pH (Amel Instrument, 334-B, Milan, Italy), and salt content [21] were determined. Concentrations of organic acids were also determined by HPLC [22]. Briefly, 1 g of cheese was diluted in 5.0 mL in ultrapure water and vigorously shaken for 20 s. After centrifugation at 3000× *g* for 15 min, the supernatant was filtered through a 0.45 μm and a 0.22 μm cellulose membrane. The HPLC system consisted of two pumps (Jasco PU1580), an auto-sampler (Waters 717 plus), and a UV-VIS detector (Waters 484) set at 210 nm. The separation was performed on a Rezex ROA column 300 mm × 7.8 mm, 8 μm (Phenomenex, Torrance, CA, USA). The mobile phase (0.5 mL/min in isocratic mode) was 0.005 N sulphuric acid. External standards (Sigma Aldrich, St. Louis, MO, USA) were used for the identification and quantification of acetic, citric, and lactic acids. Eight concentration points in triplicate were used to prepare the calibration curves. The concentrations of each compound were prepared from stock solutions by dissolving the proper quantity in 10 mL of water. The range of concentrations of citric, lactic, and acetic acid were 0.16–32.32 mM, 0.28–27.75 mM, and 0.21–41.13 mM, respectively. For each compound, the coefficients of determination (R2) were calculated and the linearity was analyzed on the basis of the relative standard deviation (R.S.D.) values for the corresponding response factors. The coefficients of determination (R2) obtained were excellent, with values better than 0.999. The R.S.D. values of the respond factor were in the range 0–5%, and were considered adequate to verify the linearity of the regression lines for analytical methods. Accuracy was determined using an added external standard. A sample of cheese was spiked in triplicate with known quantities of three of the organic acids and the percentage of recovery was calculated. Recovery was calculated from the concentration quantified using the calibration curves versus the concentration added, obtaining values close to 100% for all of them. The limit of quantification (LoQ: 0.16 mM (30.74 mg/kg), 0.28 mM (25.22 mg/kg), and 0.21 mM (12.61 mg/kg) for citric, lactic, and acetic acid, respectively) was calculated by the signal-to-noise approach [23].

### 2.8. Determination of Growth Ability of B. cereus Strains at Different Temperatures

For the subsequent tests, five isolates were selected basing on the presence of all or nearly all toxins and toxin-encoding genes. A loop of each bacterial stock was inoculated in nutrient broth tubes (Sigma-Aldrich, 70122) and incubated at 37 °C. Cells were harvested in exponential growth phase and cell concentration was determined by counting under phase-contrast microscopy (BA 310, Motic, Barcelona, E) [24]. Bacterial concentration was adjusted with sterile saline water (0.85% NaCl) to 1.5 Log cfu/mL. Suspensions were seeded on Nutrient Agar (Sigma-Aldrich, 70148), and incubated at different temperatures of 5, 7, 10, and 15 °C. Plates were monitored daily for colony formation for up to 15 days [25,26,27].

### 2.9. Harvesting of Dormant Spores

Spores of the five strains, were produced on fortified nutrient agar [28] supplemented with NaCl (5.0 mg/mL), CaCl_2_, (0.1 mg/mL), and MgSO_4_ × 7H_2_0 (2.0 mg/mL). Roux bottles containing 150 mL of the medium were inoculated with 2.0 mL of spore suspension (∼7 Log spores/mL in distilled water). After incubation at 37 °C for 10 days, flasks were stored at 4 °C for 24 h and spores were subsequently scraped from the agar with a sterile stirrer and washed five times by centrifugation (10,000 rpm for 10 min) with ice-cold sterile distilled water. To eliminate residual vegetative cells, each spore suspension was thermally treated at 80 °C for 10 min in a water bath. Finally, the suspensions were rapidly cooled in ice, washed again with ice-cold sterile distilled water, and maintained at 2–4 °C until they were diluted for the inoculation of the samples (Section 2.10). 

### 2.10. Growth Potential of B. cereus on Cheese

For performing the challenge tests, two out of the five isolates that were characterized for their ability to grow at different temperatures were selected. The cheese to be inoculated was selected to have an enumerable count of *B. cereus* and chemical-physical characteristics that may theoretically support the growth of the bacterium. Spore suspensions of these strains were diluted in sterile saline to reach a concentration of about 5 Log cfu/mL. These suspensions were mixed in equal volumes and the mixture was spread on the surface of the cheese and immediately distributed using a spatula, assuring a concentration of 2 Log cfu/g of the product. To minimize changes in product characteristics, the inoculum volume did not exceed 1% of the final weight. Blank samples, inoculated with the same volume of sterile saline solution, were also prepared. The inoculated and blank samples were then assigned to two series, incubated in parallel at 15 °C and 10 °C. The temperature was constantly recorded by Escort iLog data loggers (Escort Data Logging System Ltd., Aesch Bei Birmensdorf, Switzerland).

Samplings were performed at the inoculum (t0) and after 2, 4, and 7 d of storage at 15 °C, or after 4, 7, and 11 d of storage at 10 °C. All analyses were performed in triplicate. From each inoculated sample, 10 g of product was diluted 10-fold in chilled sterile diluent solution and submitted to the count of presumptive *B. cereus* and spores. The samples were also submitted to the enumeration of lactic acid bacteria (LAB; ISO 15214:1998; [29]). Cheese pH was also determined in triplicate.

### 2.11. Statistical Analysis

Three independent biological replicates with two technical replicates each were performed. Quantitative data were expressed as the mean ± standard deviation (SD). Prevalence data were analyzed by exact Fisher test. Data for chemical-physical characterization were analyzed by one-way Anova for comparing cheese categories. Dendrogram analysis and distance index calculation were performed applying the Sørensen-Dice coefficient. For biofilm production, the one-way Anova for independent data followed by Tukey’s HSD test was used. A two-sided *p*-value (*p*) < 0.05 was considered significant. 

## 3. Results and Discussion

In this study, the presence of *B. cereus* was investigated in various fresh and short-ripened cheeses purchased on the retail market during different sampling sessions. As reported in Table 1 (and Table S1), *B. cereus* was presumptively isolated from 12/122 of the samples (prevalence 9.8%). In all of the 12 *B. cereus*-positive samples, the microorganism was found in very low counts (from 1 Log cfu/g to 2.26 Log cfu/g), either considering vegetative cells or spores; this could indicate its presence in milk and/or a contamination during the process, and a possible mild growth in some cheeses, but without the occurrence of environmental conditions able to support the efficient growth of the pathogen; the counts obtained were far lower than those needed to exert a significant toxin production. Otherwise, an initial growth followed by sporulation would have resulted in high spore counts.

Limited information about *B. cereus* prevalence in cheeses is available in the literature; our value can be considered low when compared to the wide range (8–50%) described by other authors [30,31,32,33,34]. A higher prevalence of spores compared to vegetative cells was conceivable, owing to their persistence in milk and the environment; this hypothesis was confirmed by the data, with the detection of spores in ten of the twelve positive samples, and of vegetative cells in only three samples. 

**Figure 1:**
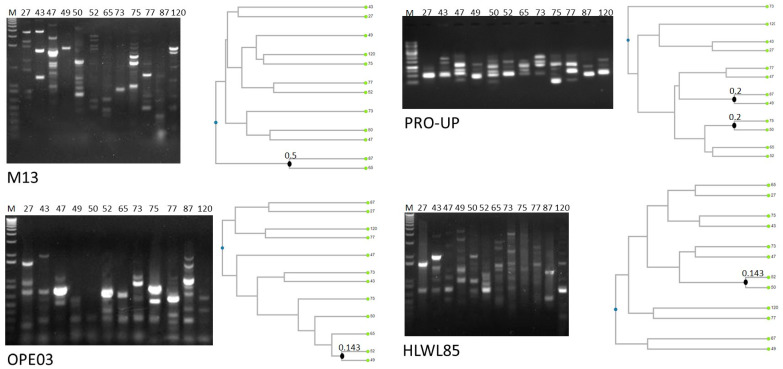
RAPD-PCR profiles and dendrogram analysis of the isolates. Closest knots obtained applying the Dice coefficient as similarity index are marketed. The strains are named with the same code used for the products from which they were isolated, as reported in Table 1.

All the isolates were identified by MALDI-TOF MS as *B. cereus sensu stricto* with scores > 2.0 (Table S2). Figure 1 shows molecular typing performed by RAPD-PCR. Statistical analysis of dendrograms generated from RAPD amplification profiles indicated that all the isolates were characterized by unique amplification patterns, and were, thus, different strains. Indeed, strains possessing similar profiles with one primer presented different patterns with the other three primers. This result was expected, since the strains were isolated from different cheeses produced by different brands and at different times, thus indicating that no specific strain circulates among different cheeses sold on the Italian market. 

The presence of *B. cereus sensu stricto* in foods may constitute a concern for human health and a serious risk for the consumer. In addition, this microorganism can cause cheese spoilage, owing to the action of its enzymes, such as phospholipases and proteases, which can affect products quality [35]. For this reason, we wondered whether the isolated *B. cereus* strains could produce virulence proteins and enzymes (Table 2). We evaluated the ability of *B. cereus* strains to produce/secrete PC-PLC, proteases, and HBL by visualizing the activity of these virulence factors on solid media containing skim milk, phosphatidylcholine, and sheep blood, respectively. All the strains were found able to produce proteases, and most secreted PC-PLC (92%). In contrast, HBL production was only detected in 42% of the strains (Table 2). These results agree with previous data showing a very high rate of PC-PLC and proteases, and a variable rate of HBL production (ranging from 20 to 90%) among *B. cereus* strains [36]. 

For the other virulence factors, no specific assays are available. Therefore, PCR reactions on DNA extracted from the strain were performed to detect genes encoding Smase, NHE components (NHE_A_, NHE_B_, NHE_C_), EntFM, BceT, CytK, and PI-PLC (Figure S1). The most represented toxin gene was *bceT* (present in 92% of the strains; Table 2). The prevalence of *bceT* obtained in this study is comparable or slightly higher to that reported in other studies [37,38]. The presence of *sph* and of the three genes *nheA*, *nheB*, and *nheC* was revealed in 75% of isolates (Table 2), a prevalence that is in line with previous data [37]. EntFM was present in 67% of the strains, a prevalence similar to that described in the literature for other dairy products [37]. Lastly, *cytK*, encoding CytK, was only present in 33% of the strains (Table 2). As previously reported, this toxin is present at low prevalence among *B. cereus* strains isolated from dairy products [39]. 

The production of microbial biofilms, complex communities in which bacteria are embedded within an extracellular matrix, may facilitate *B. cereus* survival and persistence in the food industry and dairy processing plants [35]. The analysis of biofilm formation by crystal violet assay revealed that all the isolates were able to produce biofilms (Table S3). The establishment of this microbial community upon surfaces in the dairy plant, also if not in direct contact with food, has been previously demonstrated to be the potential source of the cross- and post-processing contamination of finished products [40].

Overall, three isolates (65, 75, and 77; 25% of the strains) possessed all the virulence factors that were considered in this study, thus resulting in the strains with the highest virulence potential. While strain 52 (8% of the strains) was the only isolate that did not secrete PC-PLC (but possessed all the other virulence factors), strains 43, 47, 50, and 87 (33% of the strains) lacked two virulence factors, strains 49 and 73 (17% of the strains) lacked six virulence factors, and 120 (8%) lacked seven virulence factors (Table 2). 

To evaluate the growth ability of *B. cereus* at relatively low temperatures, five strains harboring all or nearly all the virulence factors (65, 75, 77, 52, and 87) were selected for the evaluation of growth ability at low temperatures (i.e., 5, 7, 10, and 15 °C) for 15 days. As shown in Table 3, no strains were found able to grow at temperatures between 5 and 7 °C for up to 15 days. In contrast, at 10 and 15 °C, all the strains grew, starting from day 10 and 3, respectively. These results stress the importance of temperature management during the entire production process: if during cheese purchase, transport, and domestic storage a thermal abuse sufficient to allow growth is unlikely, temperatures of 15 °C or higher are usually applied during the first phases of the cheese-making process. Thus, milk and process hygiene procedures must be applied.

A comparison of the results obtained from microbiological and chemical-physical analyses performed on the different cheese categories showed some marked differences. Considering the prevalence of *B. cereus* (Table 1), fresh and short-ripened cheeses showed higher prevalence (14%) compared to the other categories, as well as if this difference was not statistically significant. Among fresh cheeses, a higher prevalence was observed in caprino-type cheese (3/8), in robiola cheese (2/8), and in stracchino-type cheese (2/19). All these positive samples were obtained from cow milk. These cheeses are extremely perishable products, characterized by a rich microbiota, and are mainly composed of lactic acid bacteria. Primo sale, with similar characteristics, showed one positive sample, whereas no positive samples were detected in cottage cheese. 

Among cheeses falling into the “pasta filata” (spun paste) category (namely mozzarella, burrata, and stracciatella), *B. cereus* was detected in just one sample of stracciatella. This cheese includes two mixed substrates (cheese and cream), both coming from pasteurized milk/cream; thus, it is difficult to find a contamination source which can originate both from milk or from the production environment. Some prevalence data regarding Italian mozzarella cheese have been reported [31,34] with relatively high prevalence (11.1–26.8%), whereas no information is available for the other cheeses belonging to this category. Considering short-ripened cheeses, only two positive samples (one “quartirolo” and one “other” short-ripened cheese) were detected, but the number of samples in each category was low.

The fresh cheeses and short-ripened cheeses analyzed in this study included a wide range of product typologies obtained with different technologies, that could vary in their suitability as substrates for eventual *B. cereus* growth. These differences were reflected in the intrinsic characteristics of each considered product, as shown by the results obtained from the physicochemical analyses (Table 4 and Table 5). 

As different brands were considered for some of the cheese typologies, it was possible to evidence, in some cases, a wide variety in the characteristics of the samples belonging to the same product denomination. With this characterization, we aimed to supply more complete information about the fresh or short-ripened cheeses available on the market, and to correlate these intrinsic characteristics to the potential ability of *B. cereus* to replicate in these substrates. The samples had very dissimilar moisture content, with significant variability from 35% to 82%; the highest values were measured in ricotta and cottage cheese (more than 70%), followed by various “pasta filata” cheeses. When comparing the various cheese categories (Table 5), some significant differences were shown; higher moisture and Aw values, and lower salt content were detected in ricotta, pasta filata, and fresh cheeses, whereas the short-ripening period resulted in product drying; a particular case was represented by mascarpone, which is made by milk cream (thus, its low moisture content was not coupled with higher salt concentration). Considering the role of cheese salting and eventual drying, it has to be noted that *B. cereus* growth can occur when salt concentration is lower than 7.5%. None of the samples analyzed exceeded this threshold, and therefore, salt does not represent an obstacle that can act as a unique, efficient hurdle to prevent to the growth of the microorganism. The lower water activity value that allows *B. cereus* growth is 0.930; only one sample in which *B. cereus* was found (sample 35—Brie cheese) showed a value lower than this threshold (0.902). In addition to brie cheese, an evident variability in values was observed for stracchino cheese. This finding could be due to the fact that products with different production processes and ripening periods can be marketed with the same denomination. All the other samples showed values higher than the threshold, and therefore, Aw conditions were favorable to the growth of the microorganism. pH showed significantly lower values in fresh and short-ripened cheeses, while values often above 6.0 were detected in the other cheese categories, owing to specific production technologies, or to the de-acidifying action of molds (Table 3). The favorable range of pH for the growth of *B. cereus* is between 5.0 and 10.0. The measured pH values were extremely variable among samples, with a mean value of 5.64 ±0.80 and minimum and maximum values of 4.06 and 7.16, respectively. In this wide range of pH, not all cheeses analyzed are substrates in which *B. cereus* may find favorable conditions for its growth. A total of 24 samples (19.35% of the total) resulted in pH values lower than 5.0, thus representing a harsh environment for bacterial growth, except for some *Lactobacillus* strains that are used for the production of some dairy products. These were all the samples of caprino cheese, feta cheese, and cottage cheese, nearly all robiola cheese samples, and one sample of quartirolo cheese. It must be noted that six of the twenty-four samples tested positive for the presence of *B. cereus* (namely, samples 27, 49, 50, 52, 73, and 120). Therefore, in these cheese typologies, the presence of the microorganism was not associated with permissive pH conditions. A wide variability was observed in some typologies. This was particularly evident when molds were involved in cheese production, as in the case of camembert, brie, or some other similar cheeses.

The concentration of organic acids in the analyzed cheeses reflected the production technology applied: lactic acid reached significantly higher values in fresh and short-ripened cheeses (in parallel with lower pH values), whereas citric acid was mainly detected in mascarpone, ricotta, and pasta filata cheeses, that are often submitted to its addition during production; acetic acid seemed to be linked to the development of yeast and molds, as significantly higher values were detected in ripened cheeses. The concentrations were extremely variable (Table 4). Citric acid showed very variable but generally low concentrations. The values ranged from <LoQ (30.74 mg/kg) to >5000 mg/kg; the highest values were measured in some mozzarella and ricotta cheese samples. This result was expected, as citric acid is frequently added to these cheeses during the production process. Citric acid is known as a mild antimicrobial agent [41], and high concentrations are needed to act as effective single hurdles (thus modifying the sensorial characteristics of foodstuffs). For this reason, citric acid is often used in combination with other organic acids. Lactic acid can be added during the production of some cheeses, but it is mainly produced by the microbiota of fresh dairy products. Lactic acid content was extremely variable in the analyzed samples, both within and among the cheese typologies, with values from <LoQ (25.22 mg/kg) to >20,000 mg/kg. Lactic acid is considered a more efficient antimicrobial agent than citric acid, thanks to its lower pKa (3.86 vs. 3.13 of citric acid), which has a higher relative concentration of undissociated form, and possesses an inhibitory action against pathogenic bacteria [42]. Heterogeneous but generally lower concentrations of acetic acid were detected. The maximum value determined was ~2700 mg/kg, in a camembert sample. This result was expected, since acetic acid derives from fermentation processes occurring in the cheese maturation stage by yeasts and molds. Acetic acid is considered an efficient antimicrobial compound due to its ability to reduce pH, and to its relatively high pKa (4.76) causing damage to the bacterial cell walls, but also penetrating the cells [43]. The Minimal Inhibitory Concentration of organic acids towards *B. cereus* was determined in vitro by Hsiao and Siebert [44]. Values of 3680 mg/L (citric acid), 3480 mg/L (lactic acid), and 2020 mg/L (acetic acid) were defined in culture broth. These values were reached and exceeded in many cheese samples analyzed in this study, but to evaluate their real efficacy we must consider the complexity of cheese matrices, which cause a marked increase in MIC values [45,46].

**Figure 2:**
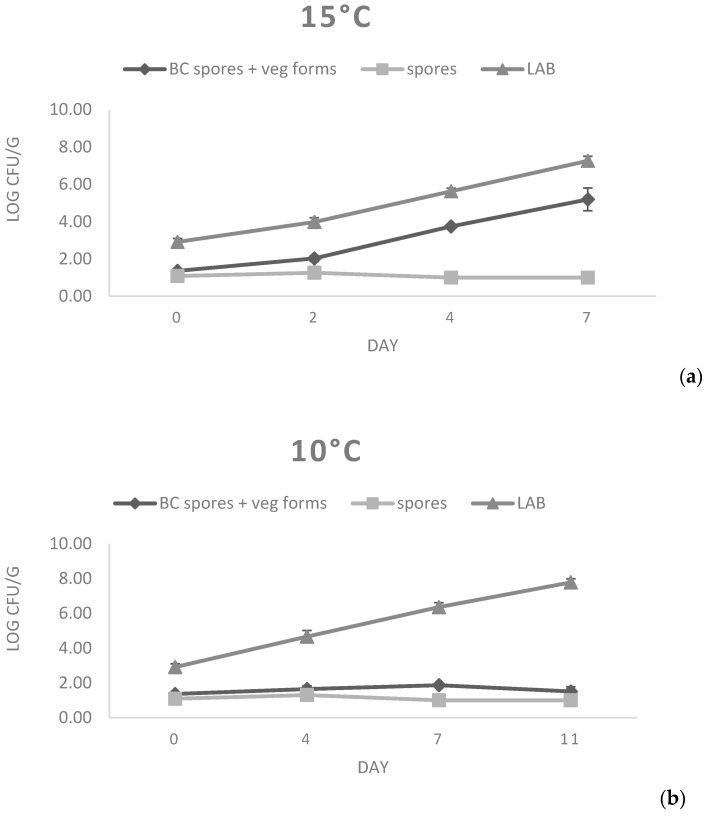
*B. cereus* growth at 15 °C (**a**) and 10 °C (**b**) on primo sale cheese.

For the evaluation of the potential growth of *B. cereus* strains on fresh cheese substrate, a challenge test was performed on primo sale cheese using a mixture of spores obtained from strains 77 and 87. Primo sale was selected as a substrate, since it presented *B. cereus* contamination (Table 1) and showed permissive chemical physical characteristics (pH = 6.34, Aw = 0.979, Salt content = 0.36%; Table 4). For the test, a mixture of strains 77 and 87 was prepared; strain 77 was selected owing to the fact that it harbored all the virulence factors (Table 3), whereas strain 87 was chosen as it had been isolated from primo sale cheese, thus mimicking natural contamination as much as possible. As evidenced in Figure 2, the number of spores quantified at both temperatures was stable over time, with only a slight decrease observed at 4 and 7 d at 15 °C that was compatible with the germination of spores. In fact, vegetative cells showed a fast growth at 15 °C. In particular, starting from a count of 1.36 Log cfu/g, an average increase of 3.83 Log cfu/g in the considered storage time (7 days) was observed. The ability to grow at 15 °C has been confirmed in previous studies, where ricotta cheese, mascarpone cheese, and pasteurized milk were determined to be good substrates for *B. cereus* growth [11,12]. In contrast, *B. cereus* strains did not multiply for up to 11 days at 10 °C, since no increase in the number of vegetative cells was found.

At both 15 °C and 10 °C, rapid growth of lactic acid bacteria was detected. In fact, starting from a mean initial count of 2.91 Log cfu/g, final counts of 7.26 and 7.78 Log cfu/g were reached at 10 °C and 15 °C, respectively. This growth justifies the decrease in pH values observed in both challenge tests, reaching mean values below 6.0 after the fourth day of storage (Table 6). 

The inhibitory activity of lactic acid bacteria towards the pathogenic or spoilage microorganisms in food matrices is a well-known phenomenon called the “Jameson effect”, and has been observed in many dairy products and non-dairy foods, where nutrients were depleted by the growth of the natural microflora, limiting the growth of other contaminant bacteria [11,47]. In this case, such an event was not observed, suggesting that the LAB population did not reach a sufficiently high enough count to play an effective role during the experimental period; higher counts could be presumably reached with longer storage (as seen in the figures, only the logarithmic growth phase of LAB growth curve was observed), but this would also result in a modification of product characteristics. Similar LAB growth curves were evidenced at the two tested temperatures, whereas the behavior of *B. cereus* was significantly different. Thus, the main inhibiting role was played by the storage temperature: as already observed in the in vitro assay, a temperature of 10 °C is near the lower growth limit for the analyzed strains. As described in previous studies [12,48], the ability of *B. cereus* to grow at a certain temperature depends on the conditions of the substrate (pH, atmosphere, etc.), and suboptimal environmental conditions (such as those present in cheese instead of growth medium) are thought to be sufficient to prevent the significant growth of the microorganism. Owing to the complexity and variability of cheese matrices, the outcomes of the challenge test could be hardly extended to other product typologies, but these results give a picture of the pathogen behavior in a favorable substrate. The risk posed by the potential growth of toxigenic *B. cereus* strains in some fresh cheeses should thus be considered in the planning of food safety management systems of dairy industries; major attention should be paid to the first cheese production phases, when the substrate and environmental conditions could be favorable for *B. cereus* growth and potential toxin production. 

## 4. Conclusions

The presence of *B. cereus* in fresh and short-ripened cheeses marketed in Italy was evidenced with relatively low prevalence and counts. Nevertheless, the presence of virulence factors among the isolated strains indicates that the production of toxins and enzymes is widespread in circulating *B. cereus* strains, suggesting the potential harmfulness of *B. cereus* contamination. Moreover, the ability to form biofilm, which was common among different strains, highlights the potential persistence of this pathogen in production lines.

The presence of vegetative cells and/or spores of *B. cereus* in different typologies of cheeses may be favored by its spore forming ability, which reduce the impact of milk pasteurization, as well as by the ubiquitous nature of this microbe.

Chemical physical characterization of the cheeses showed variable values and conditions that can be considered favorable for *B. cereus* growth in many cheese typologies. Nevertheless, these favorable conditions were rarely coupled with the detection of *B. cereus.* When considering a whole hurdle technology approach, other intrinsic factors (cheese microbiota, physical structure, etc.) would probably affect the potential growth of the pathogen. In addition, extrinsic hurdles must be considered: in this study, temperature resulted in being the main influencing parameter, as the strains isolated from cheeses did not grow at temperatures equal or below 7 °C. Thus, the usual refrigeration temperatures applied during cheese storage should be sufficient to prevent growth. 

The results obtained in this study should stress the role of the food business operator in contrasting the presence of high counts of *B. cereus* in dairy products. As milk and environmental contaminations are not completely avoidable, *B. cereus* contamination should be managed by the application of treatment parameters (pasteurization time/temperature combinations) able to inactivate *B. cereus* vegetative cells, followed by a strict application of hygiene prerequisites to limit spore diffusion on the cheeses; these include limitations in the circulation of materials and persons, the proper and frequent sanitization of equipment, and, especially for products lacking intrinsic effective hurdles, constant monitoring of storage temperatures.

## Figures and Tables

**Table 1:** *B. cereus* enumeration (total organisms and spores) in fresh and short-ripened cheeses.

Category	Product	N° Samples	N° Positive Veg.–Spores	Count Range (Logcfu/g)	Strain n°
Pasta filata	Mozzarella (C)	13	0-0		
	Mozzarella (B)	1	0-0		
	Burrata (C)	4	0-0		
	Stracciatella (C)	4	0-**1**	**1.00**	*49*
	**Subtotal**	**22**	0-**1**		
Fresh cheese	Crescenza (C)	5	0-0		
	Stracchino (C)	19	0-**2**	**1.00**	*47, 65*
	Squacquerone (C)	4	0-0		
	Caprino (C)	5	0-**2**	**1.30–1.48**	*50, 73*
	Caprino (G)	34	**1**-0	**1.00**	*120*
	Primo sale (C)	6	**1**-0	**1.00**	***87***
	Primo sale (G)	1	0-0		
	Robiola (C)	6	**1-2**	**1.00–2.26**	*27, 43*
	Robiola (G)	2	0-0		
	Cottage (C)	4	0-0		
	Fresh cheese (G)	1	0-0		
	**Subtotal**	**56**	**3-6**		
Short ripened	Feta (S + G)	3	0-0		
	Caciotta (C)	3	0-0		
	Tomino (C)	2	0-0		
	Taleggio (C)	1	0-0		
	Quartirolo (C)	1	0-**1**	**1.30**	*52*
	Pecorino (S)	1	0-0		
	Monte Veronese (C)	1	0-0		
	Other short ripened (C)	1	0-**1**	**1.00**	*75*
	Other short ripened (C + G)	1	0-0		
	**Subtotal**	**14**	0-**2**		
Mold ripened	Brie (C)	4	0-0		
	Camembert (C)	1	0-0		
	Camembert (G)	1	0-0		
	Camembert (B)	1	0-0		
	Other mold ripened (C)	4	0-0		
	Other mold ripened (G)	2	0-**1**	**1.30**	*77*
	**Subtotal**	**13**	**0-1**		
Ricotta	Ricotta (C)	13	0-0		
	Ricotta (S)	1	0-0		
	Ricotta (G)	1	0-0		
	**Subtotal**	**15**	**0-0**		
Mascarpone (C)		2	0-0		
**Total**		**122**	**3-10**		

C = cheese made by Cow milk; B = Buffalo milk; S = Sheep milk; G = Goat milk. <10 CFU/g indicates bacterial counts below the limit of quantification. Detectable loads of presumptive *B. cereus* were marked in bold.

**Table 2:** Detection of virulence factors in *B. cereus* strains.

Strain	HBL	PC-PLC	Proteases	*sph*	*nheA*	*nheB*	*nheC*	*entFM*	*bcet*	*cytK*	*plcA*
27	−	+	+	−	+	+	+	−	+	−	+
43	−	+	+	+	+	+	+	+	+	−	+
47	−	+	+	+	+	+	+	+	+	−	+
49	+	+	+	+	−	−	−	−	+	−	−
50	−	+	+	+	+	+	+	+	+	−	+
52	+	−	+	+	+	+	+	+	+	+	+
65	+	+	+	+	+	+	+	+	+	+	+
73	−	+	+	−	+	−	+	−	+	−	−
75	+	+	+	+	+	+	+	+	+	+	+
77	+	+	+	+	+	+	+	+	+	+	+
87	−	+	+	+	+	+	+	+	+	−	+
120	−	+	+	−	+	−	+	−	−	−	−

**Table 3:** Growth of *B. cereus* isolates 52, 65, 75, 77, and 87 at different temperatures.

Strain	T (°C)	Day														
		1	2	3	4	5	6	7	8	9	10	11	12	13	14	15
52	5	−	−	−	−	−	−	−	−	−	−	−	−	−	−	−
	7	−	−	−	−	−	−	−	−	−	−	−	−	−	−	−
	10	−	−	−	−	−	−	−	−	−	+	+	+	+	+	+
	15	−	−	+	+	+	+	+	+	+	+	+	+	+	+	+
65	5	−	−	−	−	−	−	−	−	−	−	−	−	−	−	−
	7	−	−	−	−	−	−	−	−	−	−	−	−	−	−	−
	10	−	−	−	−	−	−	−	−	−	+	+	+	+	+	+
	15	−	−	+	+	+	+	+	+	+	+	+	+	+	+	+
75	5	−	−	−	−	−	−	−	−	−	−	−	−	−	−	−
	7	−	−	−	−	−	−	−	−	−	−	−	−	−	−	−
	10	−	−	−	−	−	−	−	−	−	+	+	+	+	+	+
	15	−	−	+	+	+	+	+	+	+	+	+	+	+	+	+
77	5	−	−	−	−	−	−	−	−	−	−	−	−	−	−	−
	7	−	−	−	−	−	−	−	−	−	−	−	−	−	−	−
	10	−	−	−	−	−	−	−	−	−	+	+	+	+	+	+
	15	−	−	+	+	+	+	+	+	+	+	+	+	+	+	+
87	5	−	−	−	−	−	−	−	−	−	−	−	−	−	−	−
	7	−	−	−	−	−	−	−	−	−	−	−	−	−	−	−
	10	−	−	−	−	−	−	−	−	−	+	+	+	+	+	+
	15	−	−	+	+	+	+	+	+	+	+	+	+	+	+	+

**Table 4:** Range of chemical and physical characteristics (minimum and maximum) of the cheese samples.

Product Typology	Moisture %	Salt conc. %	Aw	pH	Citric Acid (mg/kg)	Lactic Acid (mg/kg)	Acetic Acid (mg/kg)
Burrata (C)	61.9–69.3	0.22–0.98	0.972–0.992	5.35–6.28	n.d.–585	1273–11,232	n.d.–871
Mozzarella (C)	59.1–74.6	0.32–0.99	0.975–0.991	5.77–6.76	<LoQ–5347	<LoQ-5946	n.d.–274
Mozzarella (B)	71.0	1.06	0.981	5.37	1111	16,469	654
Stracciatella (C)	63.8–66.4	0.32–0.72	0.974–0.992	4.77–6.58	<LoQ–1142	566–4840	205–535
Caprino (C)	58.4–71.6	0.80–0.92	0.968–0.983	4.18–4.98	n.d.–596	5170–10,156	320–593
Caprino (G)	59.3–68.4	0.45–1.00	0.980–0.981	4.34–4.35	n.d.–312	6758–7823	368–927
Cottage (C)	79.5–82.4	0.55–0.85	0.977–0.992	4.11–4.57	<LoQ–1557	4471–5999	n.d.–529
Crescenza (C)	55.8–62.1	0.79–1.14	0.965–0.984	5.17–5.43	n.d.–2026	6431–11,449	n.d.–887
Stracchino (C)	54.3–72.3	0.35–1.32	0.938–0.985	5.02–5.50	n.d.–458	6549–12,454	n.d.–1142
Primosale (C)	40.6–70.2	0.36–1.34	0.970–0.983	5.05–6.51	n.d.–2200	2001–9566	n.d.–655
Primosale (G)	43.2	0.94	0.974	5.59	<LoQ	11,531	871
Robiola (C)	54.3–65.9	0.63–1.22	0.964–0.992	4.45–5.74	156–523	3074–7790	n.d.–424
Robiola (G)	66.3–67.1	0.82–0.90	0.972–0.985	4.28–4.37	<LoQ	5052–7243	n.d.–450
Squacquerone (C)	57.3–70.1	0.68–0.89	0.954–0.978	5.27–5.45	<LoQ–400	5687–8271	<LoQ
Other fresh cheese (G)	67.4	0.91	0.975	4.42	347	8119	523
Caciotta (C)	45.9–49.3	1.54–1.85	0.962–0.967	5.43–5.47	<LoQ	9052–9437	156–491
Feta (S + G)	52.3–57.4	1.55–2.97	0.948–0.969	4.06–4.86	<LoQ–1491	15,281–20,409	n.d.–343
Monte Veronese (C)	41.8	1.32	0.969	5.55	251	13,632	521
Pecorino (S)	35.5	1.43	0.958	5.73	<LoQ	11,240	397
Quartirolo (C)	64.7	2.00	0.969	4.27	<LoQ	16,074	844
Taleggio (C)	49.5	1.48	0.970	5.78	<LoQ	7056	1703
Tomino (C)	46.2–49.8	0.98–1.32	0.984–0.990	6.43–6.49	331–3404	635–2159	274–1334
Other short-ripened cheese (C)	48.4	1.47	0.977	6.00	<LoQ	1753	2042
Other short-ripened cheese (C + G)	51.5	1.42	0.975	5.74	<LoQ	2864	2598
Brie (C)	40.3–50.0	1.50–2.14	0.902–0.979	6.03–6.83	n.d.–398	490–2007	n.d.–1193
Camembert (C)	52.7	1.58	0.982	6.88	295	2654	2440
Camembert (G)	50.8	1.49	0.981	6.20	<LoQ	2366	1438
Camembert (B)	53.2	2.05	0.979	5.64	n.d.	278	n.d.
Other mold ripened cheese (C)	39.8–49.5	1.43–1.81	0.970–0.986	6.31–7.16	<LoQ–172	1206–1443	196–1529
Other mold ripened cheese (G)	53.2–58.8	1.501.93	0.979–0.982	5.32–6.85	930–2541	<LoQ–77	690–1119
Ricotta (C)	68.6–82.2	0.23–0.48	0.967–0.986	5.94–6.95	<LoQ–3978	334–15,237	n.d.–1352
Ricotta (S)	76.3	0.23	0.984	6.18	1319	<LoQ	377
Ricotta (G)	77.6	0.23	0.978	6.83	<LoQ	1348	<LoQ
Mascarpone (C)	44.0–52.6	0.10–0.12	0.972–0.986	6.30–6.66	n.d.	407–1180	n.d.

C = cheese made with Cow milk; B = Buffalo milk; S = Sheep milk; G = Goat milk. <LoQ = values lower than the limit of quantification; n.d.: not detectable.

**Table 5:** Mean values (and standard deviations) obtained by the chemical-physical characterization of the cheese typologies. Different superscript letters within the same column indicate a significant difference (*p* < 0.01) among the cheese categories.

Product Typology	Moisture %	Salt conc. %	Aw	pH	Lactic Acid (mg/kg)	Citric Acid (mg/kg)	Acetic Acid (mg/kg)
Pasta filata	66.5 ^B^ ± 3.5	0.61 ^C^ ± 0.25	0.982 ^A^ ± 0.007	6.00 ^C^ ± 0.51	2783 ^B^ ± 4047	693 ± 1162	208 ^B^ ± 246
Fresh cheeses	63.4 ^B^ ± 7.8	0.86 ^B^ ± 0.21	0.975 ^A^ ± 0.010	5.11 ^E^ ± 0.62	7112 ^A^ ± 2399	406 ± 44	277 ^B^ ± 315
Short ripened	49.6 ^C^ ± 7.0	1.68 ^A^ ± 0.54	0.968 ^B^ ± 0.011	5.40 ^D^ ± 0.76	9586 ^A^ ± 6157	537 ± 899	806 ^A^ ± 798
Mold ripened	48.9 ^C^ ± 5.4	1.66 ^A^ ± 0.25	0.971 ^B^ ± 0.021	6.31 ^B^ ± 0.50	1790 ^B^ ± 1371	103 ± 133	1022 ^A^ ± 1082
Ricotta	76.8 ^A^ ± 3.8	0.32 ^D^ ±0.09	0.978 ^A^ ± 0.006	6.56 ^A^ ± 0.30	2817 ^B^ ± 4481	957 ± 1043	240 ^B^ ± 397
Mascarpone	48.3 ^C^ ± 6.0	0.11 ^D^ ± 0.01	0.979 ^A^ ± 0.010	6.48 ^B^ ± 0.25	203 ^B^ ± 288	1537 ± 504	0

**Table 6:** pH values of primo sale cheese samples detected during the challenge tests at 10 and 15 °C.

pH	Day	0	2	4	7	11
15 °C	mean	6.36	6.28	6.18	5.52	-
	std	0.01	0.01	0.09	0.09	-
10 °C	mean	6.36	-	6.29	5.96	5.72
	std	0.01	-	0.01	0.06	0.04

## Data Availability

Not applicable.

## Supplementary Materials

The following supporting information can be downloaded at: https://www.mdpi.com/article/10.3390/microorganisms11020521/s1, Figure S1: Amplification profiles of toxin-encoding genes; Table S1: *B. cereus* enumeration (total organisms and spores) in fresh cheeses; Table S2: Identification scores obtained by MALDI-TOF analysis on three separate colonies for each isolate; Table S3: Biofilm formation by *B. cereus* strains.
