# Supplementary material for: Prevalence, Virulence Potential, and Growth in Cheese of Bacillus cereus Strains Isolated from Fresh and Short-Ripened Cheeses Sold on the Italian Market
Source: Microorganisms. 2023 Feb 18;11(2):521. doi: 10.3390/microorganisms11020521 (PMC9964947; doi:10.3390/microorganisms11020521)
Supplement: Supplementary file 1 [file microorganisms-11-00521-s001.zip › Table S1.pdf]

Table S1. *B. cereus* enumeration (total organisms and spores) in fresh cheeses.

Product	Sample code	Total (cfu/g)	Spores (cfu/g)
Burrata (C)	3	<10	<10
	19	<10	<10
	41	<10	<10
	116	<10	<10
Mozzarella (C)	1	<10	<10
	2	<10	<10
	29	<10	<10
	31	<10	<10
	38	<10	<10
	46	<10	<10
	78	<10	<10
	79	<10	<10
	80 (no lact.)	<10	<10
	97	<10	<10
	102	<10	<10
	119	<10	<10
	121	<10	<10
Mozzarella (B)	42	<10	<10
Stracciatella (C)	23	<10	<10
	49	<10	**10**
	86	<10	<10
	110	<10	<10
Caprino (C)	25	<10	<10
	50	<10	**30**
	72	<10	<10
	73	<10	**20**
	117	<10	<10
Caprino (G)	17 (no lact.)	<10	<10
	74	<10	<10
	120	**10**	<10
Cottage (C)	21	<10	<10
	37	<10	<10
	88	<10	<10
	115	<10	<10
Crescenza (C)	6	<10	<10
	7 (no lact.)	<10	<10
	69	<10	<10
	70	<10	<10

Product	Sample code	Total (cfu/g)	Spores (cfu/g)
Primosale (C)	32	<10	<10
	34 (no lact.)	<10	<10
	48	<10	<10
	57	<10	<10
	87	**10**	<10
	111 (pepper)	<10	<10
Primosale (G)	99	<10	<10
Robiola (C)	10	<10	<10
	27	**180**	**170**
	43	<10	**10**
	58	<10	<10
	81	<10	<10
	95	<10	<10
Robiola (G)	26	<10	<10
	124	<10	<10
Squacquerone (C)	20	<10	<10
	45	<10	<10
	59	<10	<10
	60	<10	<10
Other fresh cheese (G)	92	<10	<10
Caciotta (C)	44	<10	<10
	96	<10	<10
	100	<10	<10
Monte Veronese (C)	98	<10	<10
Pecorino (S)	113	<10	<10
Quartirolo (C)	52	<10	**20**
Taleggio (C)	112	<10	<10
Tomino (C)	11	<10	<10
	101	<10	<10
Other short ripened (C)	75	<10	**10**
Other short ripened (C-G)	76	<10	<10
Brie (C)	12	<10	<10
	35	<10	<10
	93	<10	<10
	94	<10	<10
Camembert (C)	108	<10	<10
Camembert (G)	15	<10	<10
Camembert (B)	109	<10	<10

	85	<10	<10
Stracchino (C)	8	<10	<10
	9 (no lact.)	<10	<10
	22	<10	<10
	28	<10	<10
	47	<10	**10**
	61	<10	<10
	62	<10	<10
	63	<10	<10
	64	<10	<10
	65	<10	**10**
	66	<10	<10
	67	<10	<10
	68	<10	<10
	71	<10	<10
	82	<10	<10
	83	<10	<10
	84	<10	<10
	105 (no lact.)	<10	<10
	118	<10	<10
Feta (S-G)	24	<10	<10
	36	<10	<10
	107	<10	<10
Other mould ripened (C)	13	<10	<10
	14	<10	<10
	103	<10	<10
	114	<10	<10
Other mould ripened (G)	16	<10	<10
	77	<10	20
Ricotta (C)	4	<10	<10
	5	<10	<10
	30	<10	<10
	33	<10	<10
	39	<10	<10
	53	<10	<10
	54	<10	<10
	55	<10	<10
	56	<10	<10
	89	<10	<10
	90	<10	<10
	104	<10	<10
	123	<10	<10
Ricotta (S)	18	<10	<10
Ricotta (G)	91	<10	<10
Mascarpone (C)	40	<10	<10
	122	<10	<10

C=cheese made by Cow milk; B=Buffalo milk; S=Sheep milk; G=Goat milk. No lact.: cheese produced assuring the absence of residual lactose. <10 CFU/g indicates bacterial counts below the limit of quantification. Detectable load of presumptive *B. cereus* were marked in bold.
