# Supplementary material for: Prevalence, Virulence Potential, and Growth in Cheese of Bacillus cereus Strains Isolated from Fresh and Short-Ripened Cheeses Sold on the Italian Market
Source: Microorganisms. 2023 Feb 18;11(2):521. doi: 10.3390/microorganisms11020521 (PMC9964947; doi:10.3390/microorganisms11020521)
Supplement: Supplementary file 1 [file microorganisms-11-00521-s001.zip › Table S2.pdf]

Table S2. Identification scores obtained by MALDI-TOF analysis on three separate colonies for each isolate

Strain	Identification	Scores
27	*B. cereus*	2.24-2.22-2.02
43	*B. cereus*	2.43- 2.38-2.01
47	*B. cereus*	2.33-2.22-2.20
49	*B. cereus*	2.19-2.07-2.23
50	*B. cereus*	2.39-2.45-2.30
52	*B. cereus*	2.40-2.41-2-42
65	*B. cereus*	2.34-2.40-2.36
73	*B. cereus*	2.49-2.38-2.33
75	*B. cereus*	2.45-2.47-2.21
77	*B. cereus*	2.21-2.08-2.25
87	*B. cereus*	2.28-2.36-2.19
120	*B. cereus*	2.20-2.35-2.36
