# Supplementary material for: Prevalence, Virulence Potential, and Growth in Cheese of Bacillus cereus Strains Isolated from Fresh and Short-Ripened Cheeses Sold on the Italian Market
Source: Microorganisms. 2023 Feb 18;11(2):521. doi: 10.3390/microorganisms11020521 (PMC9964947; doi:10.3390/microorganisms11020521)
Supplement: Supplementary file 1 [file microorganisms-11-00521-s001.zip › Table S3.pdf]

Table S3. Biofilm formation by *B. cereus* strains.

Strain	OD_{595} (Mean ± SD)	p compared to negative control[a]
27	0.276 ± 0.068	< 0.0001
43	0.234 ± 0.071	0.0027
47	0.218 ± 0.048	0.0079
49	0.203 ± 0.028	0.0242
50	0.234 ± 0.090	0.0026
52	0.195 ± 0.049	0.0390
65	0.215 ± 0.065	0.0107
73	0.225 ± 0.013	0.0051
75	0.197 ± 0.018	0.0348
77	0.226 ± 0.073	0.0048
87	0.205 ± 0.027	0.0207
120	0.202 ± 0.078	0.0255

[a] sterile LB.
