# Supplementary figures and images for: Prevalence, Virulence Potential, and Growth in Cheese of Bacillus cereus Strains Isolated from Fresh and Short-Ripened Cheeses Sold on the Italian Market
Source: Microorganisms. 2023 Feb 18;11(2):521. doi: 10.3390/microorganisms11020521 (PMC9964947; doi:10.3390/microorganisms11020521)

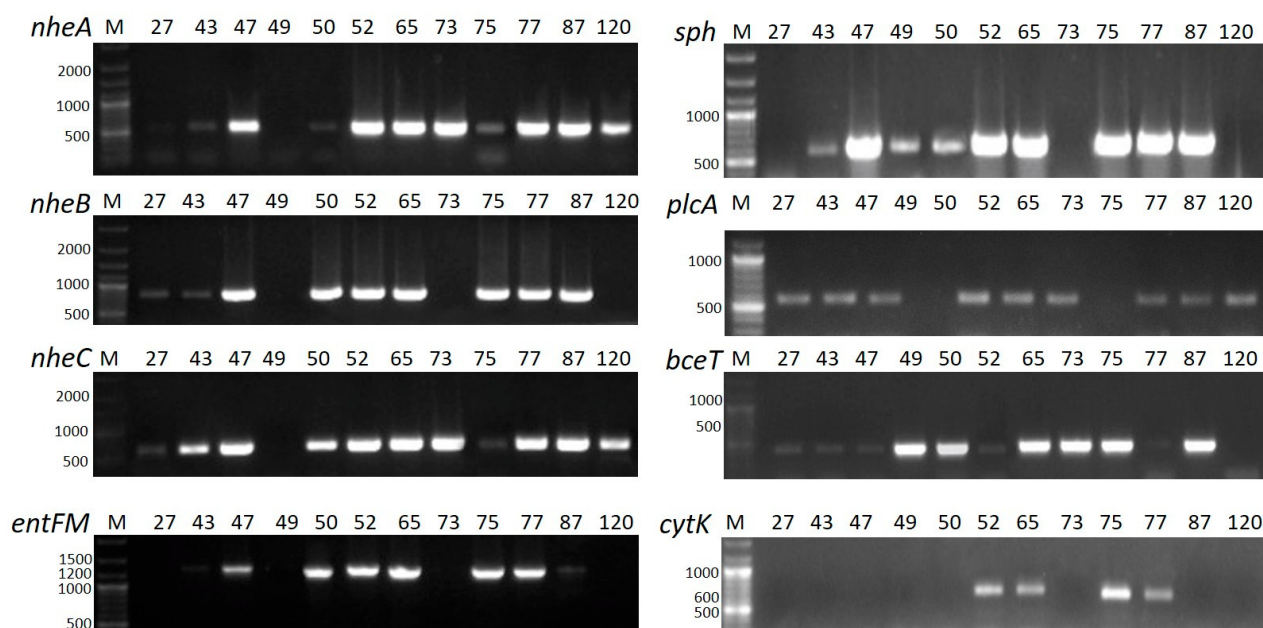

**Figure S1.** Amplification profiles of toxin-encoding genes.

Supplement: Supplementary file 1 [file microorganisms-11-00521-s001.zip › Figure S1.pdf]
